# Different exercise protocols improve metabolic syndrome markers, tissue triglycerides content and antioxidant status in rats

**DOI:** 10.1186/1758-5996-3-35

**Published:** 2011-12-19

**Authors:** José D Botezelli, Lucieli T Cambri, Ana C Ghezzi, Rodrigo A Dalia, Pedro P M Scariot, Carla Ribeiro, Fabrício A Voltarelli, Maria AR Mello

**Affiliations:** 1 Physical Education Department, São Paulo State University - UNESP, 24-A Av. 1515, Rio Claro, Zip Code:13607-331, Brazil; 2 Physical Education Department, Mato Grosso Federal University-UFMT, Brasilia Av. 1200, Cuiabá, Zip Code: 78550-000, Brazil

**Keywords:** Physical exercise, liver damage, oxidative stress, rats

## Abstract

**Background:**

An increase in the prevalence of obesity entails great expenditure for governments. Physical exercise is a powerful tool in the combat against obesity and obesity-associated diseases. This study sought to determine the effect of three different exercise protocols on metabolic syndrome and lipid peroxidation markers and the activity of antioxidant enzymes in adult Wistar rats (120 days old).

**Methods:**

Animals were randomly divided into four groups: the control (C) group was kept sedentary throughout the study; the aerobic group (A) swam1 h per day, 5 days per week, at 80% lactate threshold intensity; the strength group (S) performed strength training with four series of 10 jumps, 5 days per week; and the Concurrent group (AS) was trained using the aerobic protocol three days per week and the strength protocol two days per week.

**Results:**

Groups A and S exhibited a reduction in body weight compared to group C. All exercised animals showed a reduction in triglyceride concentrations in fatty tissues and the liver. Exercised animals also exhibited a reduction in lipid peroxidation markers (TBARS) and an increase in serum superoxide dismutase activity. Animals in group A had increased levels of liver catalase and superoxide dismutase activities.

**Conclusions:**

We concluded that all physical activity protocols improved the antioxidant systems of the animals and decreased the storage of triglycerides in the investigated tissues.

## Background

The increase in the incidence of obesity in industrialized countries in recent years has been associated with a decrease in daily energy expenditure. Studies indicate that, for the last 20 years, daily caloric intake has decreased; however, energy expenditure by means of physical activity has decreased even more. Thus, it is believed that the ongoing obesity epidemic might be more related to a reduction in physical activity than to an increase in caloric intake [1].

Obesity is associated with the appearance of systemic metabolic disorders, such as glucose intolerance, hyperinsulinemia, increased triglyceridemia, HDL cholesterol reduction and arterial hypertension. These disorders are highly associated with cardiovascular disease. This association is known as metabolic syndrome [2]. It is estimated that the prevalence of metabolic syndrome is 34% among adults and 50-60% in the population over 60 years of age in the United States, which constitutes a serious medical-social and economic problem [3,4].

Metabolic syndrome patients may also exhibit a state of chronic inflammation caused by an increased dependence on lipids as an energy source, which leads to the formation of oxygen reactive species and subsequent cell structure damage and protein structure disarray [5].

Physical activity is an important tool for the prevention of metabolic syndrome. It has been shown that aerobic training improves metabolic syndrome markers and tissue triglycerides content [6]. Also, the strength exercise can ameliorate the muscle loss and insulin sensitivity in insulin-resistant subjects [7,8]. Also, both aerobic and strength exercise can improve glucose tolerance and insulin sensitivity [6-9]. For this reason, this study sought to determine the effects of an aerobic, a strength and a concurrent (aerobic plus strength) exercise protocols on metabolic syndrome markers, lipid peroxidation markers and antioxidant enzyme activity in Wistar rats.

## Methods

### Animals and handling

Thirty-two freshly weaned Wistar rats were used in this study. Animals were kept in shared cages (four animals per cage) at a controlled temperature of 25 ± 1°C and a 12 h sleep-awake cycle. Animals had free access to water and pelleted food Labina^® ^(Purina^®^, São Paulo, Brazil). This study was performed at the Nutrition, Metabolism and Exercise Laboratory of São Paulo State University, São Paulo, Brazil. The weights of the animals were recorded weekly during the study, and the area under the curve (AUC) values were calculated using the trapezoidal rule [10] with Microsoft Excel 2007. This study was approved by the Animal Use Ethics Committee of the São Paulo State University, Biosciences Institute (CEUA), Rio Claro campus, and protocol n° 005/2010.

### Experimental groups

The Control (C) group was kept sedentary from 120 to 180 days of age. The Aerobic Training (A) group performed aerobic training 5 days per week (at 80% lactate threshold intensity) for 1 h per day from 120 to 180 days of age. The Strength Training (S) group performed strength training exercises [11] 5 days per week from 120 to 180 days of age. The Concurrent Training (AS) group performed aerobic training (at 80% lactate threshold intensity) 2 days per week (Tuesdays and Thursdays) for 1 h per day and strength training [11] 3 days per week (Mondays, Wednesdays and Fridays) from 120 to 180 days of age.

### Exercise protocol

#### Aerobic training adaptation

The sedentary (C) and aerobically trained (A and AS) groups were first adapted to the water environment. Adaptation was performedover10uninterrupted days in the same tank where the training was performed. The water temperature was kept at 31 ± 1°C [12]. The aim of adaptation was to reduce animal stress and to avoid possible physiological adaptations that might improve the physical capacity of the animals.

Rats were placed in shallow water for 10 min for three days. The water depth was then increased, as was the effort length and load (1% body weight in the form of lead ballasts placed in a Velcro^® ^backpack attached to the thorax) carried by animals. By the fourth day, animals swam for 5 min in deep water. The length of time was increased by 10 min each day until the 12^th ^day of adaptation [12].

#### Strength training adaptation

Rats were placed in shallow water tanks (31 ± 1°C) for 10 min the first two days. On the third, fourth and fifth days, the depth level was increased, and the animals were kept in the tanks for 5, 10 and 15 min, consecutively. On the sixth and seventh days, a 30% body weight overload in a Velcro^® ^"backpack" was attached to the thorax of the animals, and they were swept into the tank with shallow water. In the last three days, the animals performed 10 jumps with a 30% overload attached to the thorax, while the depth of water was progressively increased (25, 50 and 100% maximum tank capacity) [11].

#### Concurrent training adaptation

Rats were placed in shallow water (31 ± 1°C) for 10 min on the first two days. On the third, fourth and fifth days, the depth level of water was increased, and the animals were kept in the water for 5, 10 and 15 min, consecutively. On the fifth, sixth and seventh days, the animals were subjected to increased exposure times (5 min per day) in the water with a 1% body weight load attached by means of a Velcro^® ^backpack. On the last three days, the animals were subjected to strength training adaptation: on the first day, they were kept in shallow water with a load attached to the thorax (30% body weight);during the following days, they performed 10 jumps carrying this same load inside tanks filled to 50% and 100% of their maximum capacity.

#### Lactate threshold

The lactate threshold during swimming was calculated by determining the adapted "minimum lactate" test [13,14]. For this test, the animals were initially placed individually in tanks (100 cm × 80 cm × 80 cm) containing water at 31 ± 1°C. Animals carried an overload that was 13% of their body weight to induce hyperlactacidemy and were then exercised for 30 sec. After resting for 30 sec, they swam carrying the 13% load until exhaustion. After a 9 min rest, a blood sample was collected by means of a cut in the distal end of the tail to determine lactate concentration. Animals then performed exercise with progressively heavier loads [14]. The initial load was 2% of the body weight of the animal; the load was increased 0.5% every 5 min until exhaustion. After each load change, a blood sample was collected to measure lactate. The lactate minimum speed (LMS) was determined using a second-order polynomial curve adjusted to the blood lactate vs. workload curve. The blood lactate concentration was measured by spectrophotometry [15]. The lowest lactate concentration on the curve (minimum lactate) theoretically represents the maximum exercise intensity, where lactate production and removal occur in the same proportions [16].

### Physical training

#### Aerobic protocol

This protocol consisted of the animals swimming in individual tanks that contained water at 31 ± 1°C for 1 h per day, 5 days per week. Exercise was performed with the 80% individual minimum lactate intensity overload attached to the thorax of the animal.

#### Strength Protocol

Animals performed jumps in individual tanks with the water level standardized at 150% body length and a water temperature of 31 ± 1°C. Animals performed four 10-jump series with a 50% body weight overload attached to the thorax and a 1-min rest between series for 5 days per week.

#### Concurrent protocol

Animals were trained using the aerobic protocol three times a week (Mondays, Wednesdays and Fridays) and using the strength protocol twice a week (Tuesdays and Thursdays).

### Metabolic syndrome markers

Body weights, oral glucose tolerances, insulin sensitivities, blood glucose levels, lipid profiles and liver and fatty tissue triglyceride concentrations from multiple areas of the bodies of the animals were used as metabolic syndrome markers.

### Tests performed

#### Oral glucose tolerance test - oGTT

Oral GTT was performed in animals after a 12-h fast. First, a blood sample was collected from the tail end (fasting). Then, a 20% glucose solution (2 g/kg body weight) was administered to rats by a polyethylene gastric tube. Blood samples were collected after 30, 60 and 120 min by heparinized capillary tubes calibrated for 25 μL to establish the glucose and insulin concentrations. The blood glucose concentration was measured using the glucose oxidase method [17]. Results were analyzed by establishing the serum glucose AUG values by means of the trapezoidal rule [10] using Excel 2007.

#### Insulin tolerance test-ITT

Insulin sensitivity was assessed by means of a subcutaneous insulin tolerance test. It consisted of subcutaneous administration of Humalog^® ^(Lilly^®^, São Paulo Brazil) (insulin, 300 mU/Kg body weight), followed by blood sampling at 0, 30, 60 and 120 min. The blood glucose removal rate (KITT), which was expressed as %/minute, was calculated using the formula (0.0693/t/2) x100. The t/2 blood glucose was calculated by the least-square analysis of the curve of serum glucose contents, as long as a linear decrease after insulin administration was evident [18].

#### Biological material

Forty-eight hours after the last *in vivo *test, animals were sacrificed by intraperitoneal anesthesia (sodium thiopental, 40 mg/kg body weight). Two blood samples were collected via the liver portal vein. One sample was used to measure glucose, triglycerides, HDL cholesterol, LDL cholesterol and total cholesterol concentrations by means of a commercial kit (Laborlab^®^, São Paulo, Brazil) [19]. The other sample was used to assess TBARS concentration and to estimate catalase and superoxide dismutase (SOD) activities. Two liver samples were collected, one to determine the triglyceride concentration and the other to assess oxidant status biomarkers (TBARS concentration and catalase and SOD activities). Finally, fatty tissue was removed from the subcutaneous, retroperitoneal and mesenteric areas to assess the triglyceride concentrations.

### Oxidant Status Markers in the Liver

#### Antioxidant System Biomarkers

##### Catalase

To assess catalase activity, liver tissue samples (100-150 mg) were placed in Eppendorf-like tubes (1.5 mL)containing 1 mL of frozen phosphate buffer saline (PBS) and were subjected to sonication and centrifugation at 10,000 rpm for 5 min. The supernatant was separated and stored at -20°C for later analysis. Activity assays were performed by adding 50 mM phosphate buffer and 10 mM hydrogen peroxide (H_2_O_2_) to samples [20]. The drop in absorbance values by H_2_O_2 _spectrophotometry was followed. The calculation of catalase activity was performed using the equation (2.3/Δt). (a/b).(log A_1_/A_2_), where a is the volume of hemolysate in the bucket, b is the total bucket volume, A_1 _is the absorbance value at t = 0 and A_2 _is the absorbance value at the final time point (which, in our case, was 15 sec after the onset of the reaction) [20].

##### Superoxide dismutase (SOD)

To assess superoxide dismutase activity, samples of liver tissue were washed in PBS, pH 7.4, containing 0.16 mg/mL heparin, to remove blood cells. Then, tissue was homogenized (on ice) in 1 mL of 20 mM 4-2-hydroxyethyl-1-piperazineethanesulfonic acid (HEPES) buffer, pH 7.2, containing 1 mM ethylene glycol tetra-acetic acid (EGTA), 210 mM manitol and 70 mM sucrose. Next, the tissue was centrifuged at 10,000 × g for 15 min at 4°C, and the supernatant was stored at -20°C to measure total SOD (both cytoplasmic and mitochondrial levels). Measurements were performed using a commercial kit Cayman^® ^(Michigan, US). A tetrazolium salt was used to detect superoxide radicals generated by xanthine-oxidase and hypoxanthine. In this procedure, one SOD unit is defined as the amount of enzyme needed to exhibit 50% dismutation of superoxide radicals. This assay measures all three SOD types (Cu/Zn^-^, Mn^- ^and Fe^-^SOD) [21].

#### Lipid Peroxidation Biomarkers

##### Concentration of thiobarbituric acid reactive substances (TBARS)

The method for TBARS assessment consisted of analyzing the final products of lipid peroxidation (i.e., lipid peroxides, malonaldehyde and other low-molecular-weight aldehydes) that form Schiff bases upon reacting with 2-thiobarbituric acid (TBA). To assess TBARS, samples (100-150 mg) were placed in plastic tubes (RIA-type) containing 1.5 mL of cold 0.05 N phosphate buffer and were homogenized in a Polytron and centrifuged for 5 min at 10,000 rpm. The supernatants were separated, complexes were stained and their concentrations were determined by spectrophotometry at 5 nm [22].

### Statistics

Results were analyzed statistically using one-way analysis of variance (ANOVA). When indicated, a Bonferroni contrast test was applied at the 5% significance level.

## Results

**Figure 1:**
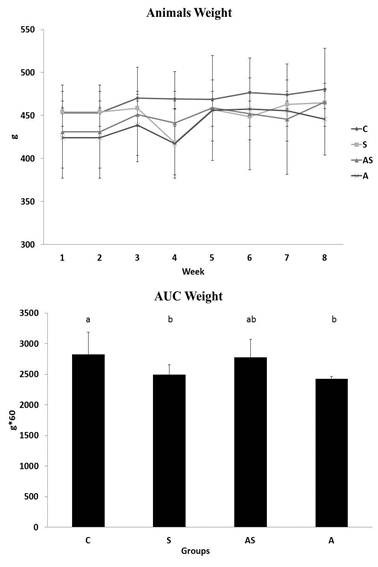
**Body weights during the study and the body weight AUC values at the end of the study**. C = Control; S = Strength Training; AS = Concurrent Training; A = Aerobic Training. n = 8 animals per group. Results are expressed as the means ± standard deviation. Different letters indicate the significant differences between groups (p ≤ 0.05).

Figure 1 shows the body weight measurements during the study and the AUC values at the end of the study. At the end of the study, A and S groups exhibited reductions in the AUC values compared to the C group.

**Figure 2:**
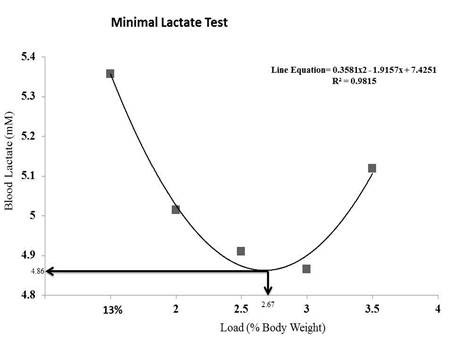
**Lactacidemia and lactate threshold equivalent overload of one animal during the minimum lactate test**. C = Control; S = Strength Training; AS = Concurrent Training; A = Aerobic Training. n = 8 animals per group. Results are expressed as the mean ± standard deviation. Different letters indicate the significant differences between groups (p ≤ 0.05).

Figure 2 shows the blood lactate and the overload values at the lactate threshold during the minimum lactate test. The results for one animal are shown as an example. For this animal, the lactate threshold was obtained with a 2.6% body weight load and a 4.86 mM lactacidemia value.

Table 1 shows the values of the areas under the serum glucose curves (AUG) during oral glucose tolerance test. No difference was found between the groups.

**Table 1:** Blood glucose (mg/dl) and glucose areas under the curve (AUG) values during the oral glucose tolerance test (oGTT)

Serum Glucose and AUG					
**Groups**	**T0**	**T30**	**T60**	**T120**	**AUG**

**C**	80.2 ± 6.0	105.8 ± 13.8	102.1 ± 6.7	79.2 ± 8.8	11354 ± 676
**A**	91.1 ± 8.9	94.0 ± 9.9	97.3 ± 8.1	92.6 ± 10.9	11343 ± 1129
**AS**	93.8 ± 8.1	95.9 ± 6.4	94.7 ± 7.5	94.1 ± 6.5	11371 ± 850
**S**	93.4 ± 6.3	93.3 ± 6.2	92.2 ± 6.5	87.7 ± 5.0	10981 ± 727

C = Control; S = Strength training; AS = Concurrent Training; A = Aerobic Training. n = 8 animals per group.

Table 2 shows the glucose removal rates during the insulin tolerance test. No differences were found between the groups.

**Table 2:** Glucose removal rates (%.min^-1^-KITT) after the insulin tolerance test (ITT)

Groups	KITT
**C**	1.04 ± 0.31
**S**	0.9 ± 0.3
**AS**	0.9 ± 0.2
**A**	0.6 ± 0.2

C = Control; S = Strength training; AS = Concurrent Training; A = Aerobic Training. n = 8 animals per group. Different letters indicate significant differences between groups (p ≤ 0.05).

Table 3 shows glucose, triglyceride, HDL cholesterol, LDL cholesterol and total cholesterol serum concentrations at the end of the study. Animals in groups A and S exhibited higher HDL cholesterol concentrations compared to group C.

**Table 3:** Glucose, triglycerides, HDL cholesterol, LDL cholesterol and total cholesterol (mg/dl) levels in the sera of the animals at the end of the study

Parameter	C	S	SA	A	
**Glucose**	98.8 ± 9.9^a^	90.0 ± 5.2^a^	88.7 ± 3.0^a^	93.0 ± 4.1^a^	
**Triglycerides**	182.8 ± 40.9^a^	174.0 ± 46.3^a^	124.5 ± 14.4^a^	163.8 ± 31.7^a^	
**HDL-Cholesterol**	42.7 ± 5.3^a^	54.4 ± 7.8^b[p < 0.05]^	53.1 ± 4.6^ab^	47.4 ± 2.2^b[p < 0.05]^	
**LDL-Cholesterol**	62.9 ± 3.9^a^	70.4 ± 6.5^ab^	70.0 ± 4.7^b[p < 0.05]^	71.1 ± 7.5^ab^	

C = Control; S = Strength Training; AS = Concurrent Training; A = Aerobic Training. n = 8 animals per group. Results are expressed as the means ± standard deviation. Different letters indicate the significant differences between groups (p ≤ 0.05).

**Figure 3:**
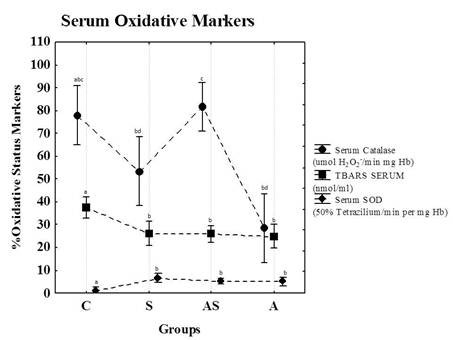
**Catalase and SOD activities and TBARS concentrations in animal serum at the end of the study**. C = Control; S = Strength Training; AS = Concurrent Training; A = Aerobic Training. n = 8 animals per group. Results are expressed as the means ± standard deviation. Different letters indicate the significant differences between groups (p ≤ 0.05).

Figure 3 shows the catalase and superoxide dismutase activities and TBARS concentrations in the sera of animals at the end of the study. All exercised groups (A, AS and S) exhibited increases in SOD activities and decreases in TBARS concentrations. Moreover, group A exhibited a reduction in catalase activity compared to groups AS and C.

**Figure 4:**
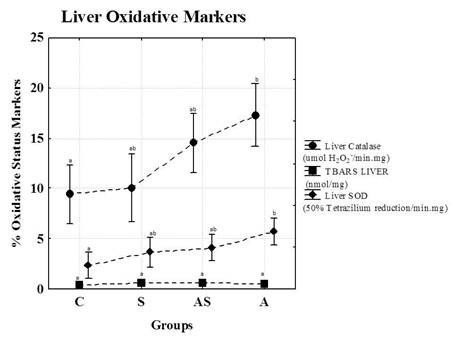
**Catalase and SOD activities and TBARS concentrations in animal livers at the end of the study**. C = Control; S = Strength Training; AS = Concurrent Training; A = Aerobic Training. n = 8 animals per group. Results are expressed as the means ± standard deviation. Different letters indicate the significant differences between groups (p ≤ 0.05).

Figure 4 shows the catalase and SOD activities and TBARS concentrations in the livers of animals at the end of the study. Animals in group A exhibited increase sin catalase activity in the liver compared to the other groups. Animals in group A also exhibited increases in SOD activity compared to group C.

Table 4 shows the triglyceride levels in the liver and in the mesenteric, retroperitoneal and subcutaneous fatty tissue at the end of the experiment. Exercised animals (groups A, AS and S) had reduced levels of triglycerides in the liver and fatty tissues in all three areas.

**Table 4:** Concentrations of triglycerides in the liver and fatty tissue in the mesenteric, retroperitoneal and subcutaneous areas at the end of the study

Triglycerides (mmol/mg)	C	S	AS	
**Liver**	13.5 ± 3.5^a^	9.1 ± 1.2^b^[^p < 0.05^]	9.0 ± 2.8^b^[^p < 0.05^]	9.9 ± 1.5^b^[^p < 0.01^]
**Mesenteric**	78.6 ± 3.0^a^	60.1 ± 6.0^b^[^p < 0.01^]	57.4 ± 15.1^b^[^p < 0.01^]	51.2 ± 12.0^b^[^p < 0.001^]
**Subcutaneous**	140.4 ± 24.0^a^	82.2 ± 6.3^b^[^p < 0.01^]	95.5 ± 12.5^b^[^p < 0.01^]	85.2 ± 10.0^b^[^p < 0.05^]
**Retroperitoneal**	101.5 ± 27.0^a^	55.5 ± 10.5^b^[^p < 0.01^]	42.0 ± 3.5^b^[^p < 0.01^]	50.5 ± 2.0^b^[^p < 0.01^]

C = Control; S = Strength training; AS = Concurrent Training; A = Aerobic Training. n = 8 animals per group. Different letters indicate significant differences between groups (p ≤ 0.05).

## Discussion

The reduction in daily energy expenditure is strongly correlated with the abrupt increase in the number of obese individuals [23]. Physical exercise is an important tool for controlling body weight [24]. In this study, animals in groups A and S exhibited reductions in the body weight AUC values compared to group C. During physical exercise, the skeletal muscle might increase its energy expenditure up to 100 times during physical exercise [25]. Minimum lactate (ML) intensity corresponds to approximately 77% of the maximum oxygen consumption (VO_2_; VO_2_max, for maximum value). In our study, animals in groups A and AS were subjected to training at 80% ML intensity, corresponding to approximately60% of VO_2_max [26]. The energy expenditure during long duration exercise performed at moderate intensity is obtained mostly by lipid mobilization, followed by carbohydrates and finally by proteins. Triglyceride mobilization might explain the weight loss of endurance-trained animals at the end of the study. Animals from group S might have responded to the increase in energy expenditure and muscular hypertrophy caused by this type of high-intensity training [27].

Insulin sensitivity and glucose tolerance are significant tests to determine the efficiency of carbohydrate metabolism. In the strength training exercises used in this study (group S), lactic glycolytic energy predominates. In this system, glucose captured by cells is degraded into two acetyl-CoA molecules that are transformed into lactate, supplying two ATP molecules [28]. This reaction is a powerful activator of carbohydrate metabolism and glucose capture by glucose transporters-4 (Glut-4). According to the results described in Table 2, there were no significant differences between the exercised groups and control in this regard. However, the glucose tolerance and insulin sensitivity values found in the exercised groups approach the values found in other studies [11,29]. Moreover, no differences were seen in the blood glucose levels of animals at the end of the study, suggesting that no alteration of carbohydrate metabolism occurred.

The lipid metabolism levels of animals were altered. There were increases in blood HDL cholesterol in groups A and S compared to the control group. Moreover, exercised animals exhibited reductions in the concentrations of triglycerides in the liver and fatty tissue in all investigated areas compared to the control. An increase in energy expenditure might explain the reductions in the concentrations of these lipids. During exercise lasting 1 to 2 h, intramuscular triglycerides are consumed, and the lipolysis mechanism is activated in the fatty tissue that supplies carbon skeletons for physical exercise. Alternatively, triglycerides might be re-esterified and stored in the muscle tissue for future use [30]. During strength exercise, there might be an increase in energy expenditure and basal metabolism due to the muscular hypertrophy [28] developed by this type of training [11]. The reduction of liver lipids in group A might be related to an improvement in liver function and the subsequent increase in HDL cholesterol production. Several studies suggest an improvement in the lipid profiles in endurance-trained rats [31-34]. The reductions of triglycerides were significant in all fatty tissue areas investigated in the trained animals. An increase in energy expenditure caused by physical training and possible metabolic alterations might be the major cause behind the improvements in the lipid profiles of rats. Similar to previous studies involving different exercise protocols [35-37], this finding shows that at different intensities, volume and energy predominance, physical exercise is an important tool in the fight against obesity.

Another beneficial effect of physical training that was observed is related to the concentrations of TBARS lipid peroxidation markers in the blood of exercised animals (groups A, AS and S). This finding is interesting because an increase in lipid oxidation is also seen in obesity. However, in this condition, lipid peroxidation is caused by the increased production of free radicals [38,39]. In the long run, this imbalance in reactive oxygen species might make the antioxidant system unable to reduce free radicals, causing its failure, decreasing its activity and blocking its protective role [40]. Physical exercise might have improved the efficiency of lipid oxidation and the antioxidant status, which was diagnosed by means of superoxide dismutase activity in animal livers in groups A, AS and S and catalase activity in the animal livers in group A. These enzymatic antioxidants are present in all mammalian cells and are associated with the reduction of O^-^and H_2_O_2_^- ^in the respiratory chain [41].

Maintenance of the antioxidant mechanism and a decrease in the structural damage caused by reactive oxygen species are very important for obese individuals. A steady high-level reactive oxygen species state could lead to cell structural damage, which, in turn, increases the concentrations of inflammatory markers such as TNF-α, interleukin 6 and interferon-γ and decreases the concentrations of adiponectin and anti-inflammatory interleukins [42,43]. Maintenance of inflammation leads to the damaging effects present in metabolic syndrome, such as endothelial dysfunction and insulin resistance [44]. Peroxidation of blood LDL cholesterol allows atheromatous plaques to accumulate on the endothelial walls, which might eventually detach and block blood flow in important small-caliber blood vessels, causing cerebrovascular accidents, myocardial ischemia and infarction [45].

Inflammatory markers decrease insulin sensitivity by causing dysfunction of insulin receptor substrate-1 (IRS-1) and insulin receptor substrate-2 (IRS-2), decreasing glucose capture and consequently increasing the dependence on lipid metabolism, thus creating a vicious circle [46]. Physical exercise might interrupt the full chain of events and make this unhealthy status regress [6]. Physical exercise might also increase the efficiency of lipid oxidation and the activity of anti-oxidizing enzymes.

In conclusion, physical exercise was efficient with respect to the following: reducing the storage of triglycerides in animal livers and fatty tissue; decreasing the body weights of animals in groups A and S; decreasing the lipid profiles of animals in group A; significantly increasing the activities of the anti-oxidizing system; and reducing the concentrations of lipid peroxidation markers. Further studies are needed to identify other physical activity intensity zones that can achieve improvement in these markers.

## Competing interests

The authors declare that they have no competing interests.

## Authors' contributions

JDB, was responsible for experimental design, data collection, statistical analysis and preparation of the manuscript. LTC, ACG, RAD, PPMS and CR were responsible for data collection. FAV was responsible for collecting data and preparing the manuscript. MARM was responsible for experimental design, coordination of research and preparing the manuscript. All authors read and approved the manuscript.
